# Efficacy predictors of omalizumab in Chinese patients with moderate-to-severe allergic asthma: Findings from a *post-hoc* analysis of a randomised phase III study^☆^

**DOI:** 10.1016/j.waojou.2020.100469

**Published:** 2020-11-24

**Authors:** Jing Li, Changzheng Wang, Chuntao Liu, Jian Kang, Lingfei Kong, Yijiang Huang, Shuang Liu, Mao Huang, Lu Wang, Robert Fogel, Xavier Jaumont, Jing Yang, Nanshan Zhong

**Affiliations:** a Allergy and Clinical Immunology Department, State Key Laboratory of Respiratory Disease, The First Affiliated Hospital, Guangzhou Medical University, Guangzhou, China; b Department of Respiratory Disease, Xinqiao Hospital, Third Military Medical University, Chongqing, China; c Department of Respiratory Disease, West China Hospital, Sichuan University, Sichuan, China; d Institute of Respiratory Disease, The First Hospital of China Medical University, Shenyang, China; e Department of Respiratory Disease, Hainan General Hospital, Hainan Clinical Medicine Research Institution, Hainan, China; f Department of Respiratory Disease, Beijing Anzhen Hospital, Capital Medical University, Beijing, China; g Department of Respiratory Disease, The First Affiliated Hospital with Nanjing Medical University, Nanjing, China; h Beijing Novartis Pharma Co. Ltd., Beijing, China; i Novartis Pharmaceuticals Corporation, East Hanover, NJ, USA; j Novartis Pharma AG, Basel, Switzerland

**Keywords:** Omalizumab, Asthma, Allergic, Immunoglobulin E, Eosinophils, China, ACQ, Asthma Control Questionnaire, ANCOVA, Analysis of covariance, AQLQ, Asthma Quality of Life Questionnaire, CMH, Cochran-Mantel-Haenszel, EOS, Eosinophil, FAS, Full analysis set, FCεR1, High affinity IgE binding receptor 1, FEV1, Forced expiratory volume in 1 s, GETE, Global Evaluation of Treatment Effectiveness, ICS, Inhaled corticosteroid, IgE, Immunoglobulin-E, IL-5, Interleukin-5, LABA, Long-acting β agonist, LSM, Least squares mean, LSM-TD, Least squares mean treatment differences, OMA, Omalizumab, PAR, Perennial allergic rhinitis, PBO, Placebo, PEF, Peak expiratory flow, PK, Pharmacokinetic, PD, Pharmacodynamics, QoL, Quality of life, RAST, Radio-allergosorbent test, RMU, Rescue medication use, SAR, Seasonal allergic rhinitis, ULOQ, Upper limit of quantification

## Abstract

**Background:**

Omalizumab has demonstrated efficacy as an add-on therapy in Chinese patients with moderate-to-severe allergic asthma. This *post-hoc* analysis assessed the potential predictors for the efficacy of omalizumab in these patients.

**Methods:**

A *post-hoc* analysis was performed on a Phase III, randomised, controlled study conducted in Chinese patients with moderate-to-severe persistent allergic asthma (NCT01202903). We evaluated if levels of pre-treatment serum total immunoglobulin-E (IgE) and blood eosinophil (EOS), asthma severity, allergen profile, history of perennial allergic rhinitis (PAR), and free IgE level during omalizumab treatment were predictive of omalizumab's efficacy.

**Results:**

This analysis included 608 patients (omalizumab, N = 306; placebo, N = 302). Improvements in forced expiratory volume in 1 s (FEV_1_), standardized Asthma Quality of Life Questionnaire (AQLQ), Asthma Control Questionnaire (ACQ), and Global Evaluation of Treatment Effectiveness (GETE) scores with omalizumab treatment compared with placebo were observed in patients with baseline IgE levels ≥76 IU/mL (irrespective of the EOS count). Relatively greater improvements with omalizumab treatment was also noted in patients with both moderate or severe allergic asthma (regardless of asthma severity), and patients sensitised to >3 allergens and with a history of PAR. All patients who were treated with omalizumab achieved free IgE levels below 50 ng/mL by Week 1. Similar clinical outcomes were observed in the subset of patients who achieved free IgE levels of <25 and ≥ 25 ng/mL.

**Conclusions:**

In Chinese patients with moderate-to-severe allergic asthma, baseline IgE and allergen profile (number/PAR history) are potential predictors of treatment response to omalizumab.

**Trial registration:**

NCT01202903 (www.clinicaltrials.gov).

## Introduction

The prevalence of asthma has increased in China over the past several decades.^1^^,^^2^ The overall prevalence of asthma in China is estimated at 4.2% in the population aged ≥20 years, representing 45.7 million Chinese adults.^3^ Of those, approximately 6.0% and 30.3% of asthmatic patients in China were categorized as having severe^4^ and moderate^5^ asthma, respectively. Asthma is a heterogeneous disease with several phenotypes, including allergic asthma and eosinophilic asthma.^6^ More than 50% of asthmatic patients in China are sensitised to allergen(s), with house dust mites being the most prevalent.^7^

Omalizumab binds to immunoglobulin-E (IgE) at the same site as the high affinity IgE binding receptor (FCεR1) to interrupt the allergic cascade.^8^ Omalizumab therapy results in reduced free IgE levels in the blood, reduced FcεRI expression on basophils, and altered IgE-mediated basophil activation (including reduced numbers of FcεRI required for activation via IgE crosslinking), and reduced allergen-mediated histamine release.^9^ Studies suggested that omalizumab is able to reduce the excessive production of IgE in patients with atopic allergic asthma over time, which in turn may affect the progression of asthma.^10^^,^^11^

A Phase III 24-week randomised clinical trial (NCT01202903) was conducted, comparing the effect of add-on omalizumab versus placebo in Chinese patients with moderate-to-severe persistent allergic asthma. The study demonstrated that treatment with omalizumab improved morning peak expiratory flow (PEF) (*P* = 0.062), forced expiratory volume in 1 s (FEV_1_) (P = 0.001), standardized Asthma Quality of Life Questionnaire (AQLQ) (*P* < 0.001), and Asthma Control Questionnaire (ACQ) (*P* = 0.002) scores at 24 weeks post-treatment compared to placebo.^12^ In China, omalizumab was approved for the treatment of moderate-to-severe persistent allergic asthma (with inadequately controlled symptoms despite inhaled corticosteroid plus long-acting β agonist [ICS + LABA] therapy) in adolescent and adult patients (aged ≥12 years) in 2017, and children (aged ≥6 years) in 2018.^13^

Although omalizumab treatment has been shown to be effective in patients with moderate-to-severe allergic asthma, only a few studies have evaluated predictors of efficacy (asthma exacerbation, lung function, AQLQ, physician's overall assessment).^14^, ^15^, ^16^, ^17^, ^18^ We have conducted this *post-hoc* analysis to further assess the predictors for the efficacy of omalizumab in Chinese patients with moderate-to-severe asthma and sensitised to common allergens. The specific aims were to: (1) determine if the efficacy of omalizumab differs between patients with different levels of pre-treatment serum eosinophil as well as total and free IgE, asthma severity, allergen profile, and history of perennial allergic rhinitis (PAR); (2) examine changes in levels of circulating total and free IgE during omalizumab treatment.

## Materials and Methods

### Study design and conduct

A *post-hoc* analysis on a 24-week, Phase III, double-blind placebo-controlled, randomised, parallel-group study, conducted across 42 centres in China in patients with uncontrolled moderate-to-severe persistent allergic asthma (clinical trial registry number: NCT01202903) was performed. See Supplemental Appendix 1 for the list of study centres and investigators, and Supplemental Appendix 2 for the inclusion and exclusion criteria. The study randomised 616 patients (1:1) to receive either placebo or omalizumab (dose and frequency according to the omalizumab dosing table) based on the patient's serum total-immunoglobulin E (IgE) level and body-weight at screening visits. It was designed to achieve an average serum free IgE of 25 ng/mL (10.42 IU/mL), with 95% patients below 50 ng/mL [20.83 IU/mL]), a level associated with clinical improvement.^19^^,^^20^ The design of the study has been reported previously.^12^ Omalizumab was shown to have a good safety profile in patients with moderate-to-severe persistent allergic asthma in the main study, and further safety assessment was not conducted during this particular analysis.^12^ The study was conducted in accordance with the current Good Clinical Practice, and the protocol was approved by Independent Ethics Committee or Institutional Review Board for each centre. Written informed consent was obtained from each enrolled patient.

### Study endpoints

The endpoints assessed in this *post-hoc* analysis include forced expiratory volume in 1 s (FEV_1_%) predicted, Asthma Control Questionnaire (ACQ) scores, standardized Asthma Quality of Life Questionnaire (AQLQ) scores, and Global Evaluation of Treatment Effectiveness (GETE) responder status at Week 24 after treatment with omalizumab or placebo. FEV_1_ values were captured at all study visits using spirometry. The standards of European Community for Coal and Steel^21^ were applied to calculate the FEV_1_% predicted values and adjusted using local conversion factor as previously reported.^22^^,^^23^ ACQ is a questionnaire consisting of 7 questions assessing symptoms, airway calibre and rescue β-agonist use,^24^ and was completed at Weeks 1, 16, and 24. AQLQ is a 32-item disease-specific questionnaire designed to measure functional impairments that were most important to patients with asthma;^25^ it was assessed at Weeks 1, 16 and 24. GETE is an assessment of asthma symptom control and overall response to asthma treatment,^26^ and it was performed by both investigator and patient, each using the same 5-point scale, at Weeks 16 and 24. The GETE scale ranges are as follows: excellent, good, moderate, poor, and worsening; a good or excellent response on the 5-point scale indicates that a patient has responded to treatment.

### Study assessment

For analysis of the predictor value of baseline serum IgE and blood eosinophil (EOS) level towards treatment outcome, the full analysis set (FAS) population was divided into 4 subgroups based on baseline EOS and IgE levels. IgE ≥76 IU/mL (182.40 ng/mL) was chosen as the cut-off point as studies have shown that patients with pre-treatment total IgE ≥76 IU/mL (182.40 ng/mL) were more likely to experience clinically meaningful benefit from omalizumab,^14^ while EOS ≥300 cells/μL was chosen as the cut-off point as patients with these levels have previously been shown to predict responsiveness to treatment with agents that target human interleukin-5 (IL-5).^27^^,^^28^ FEV_1_% predicted, ACQ score, AQLQ score, and GETE scores, were assessed in these patients at Weeks 16 and 24. For the analysis of the predictor value of asthma severity towards treatment outcome, FAS patients were categorized as moderate or severe asthmatics. ACQ score, AQLQ score, and GETE scores were assessed in these patients at Weeks 16 and 24. For the analysis of the predictor value of baseline allergen profiles towards treatment outcome, FAS patients were divided into subgroups characterized by baseline number of allergens, types of common allergens (top 4 allergens reported in the population in the analyses),^29^ and history of PAR. The outcomes assessed included ACQ score, AQLQ score, and GETE scores at Week 24**.** The blood sample of patients in the pharmacokinetic/pharmacodynamics (PK/PD) subsets were collected at baseline, Week 1, and Week 24 and analysed for concentration of total and free IgE. For free IgE, the upper limit of quantification (ULOQ) is 150 ng/mL (62.50 IU/mL), and value above the ULOQ is set to 150 ng/mL (62.50 IU/mL). To investigate the association between IgE suppression and treatment outcome, patients treated with omalizumab in the PK/PD subsets were categorized by free IgE levels (<25 or ≥25 ng/mL [<10.42 or >10.42 IU/mL]) at Week 1 and Week 24; FEV_1_% predicted, ACQ score, AQLQ score, and GETE scores were assessed at Week 24 (see Supplemental Appendix 3 for details of the subgroup analysis).

### Statistical analysis

The FAS and PK/PD population consisted of all randomised patients who received at least 1 dose of study medication, and patients who received at least 1 dose of omalizumab and had evaluable plasma concentration data, respectively.

For analysis of the predictor value of baseline serum IgE and blood EOS level, asthma severity, and baseline allergen profiles towards treatment outcome, the changes from baseline for predicted FEV_1_%, AQLQ, or ACQ scores at the end of the 24-week treatment period were analysed using analysis of covariance (ANCOVA). To investigate the association between free IgE suppression and treatment outcome, the changes from baseline for predicted FEV_1_%, AQLQ, and ACQ scores at the end of the 16- or 24-week treatment periods stratified by Week 1, and 24 free IgE values were compared using Student's t-test. A two-sided hypothesis test was conducted at an α-level of 0.05 for all endpoints. The investigator- and patient-reported GETE scores were analysed using the Cochran-Mantel-Haenszel (CMH) test, tested for treatment comparison with respect to responder rate (proportion of patients achieving excellent/good response).

## Results

### Patients

An earlier report detailed the study design and patient disposition for the study.^12^ In total, 608 patients were included in the FAS population (omalizumab, N = 306; placebo, N = 302), while 124 patients were included in the PK/PD subset (omalizumab, n = 60; placebo, n = 64). FAS patients in both treatment arms were well balanced in demographic and baseline characteristics. Of note, the mean total IgE, EOS level, baseline AQLQ score, ACQ score, FEV_1_% predicted mean values were balanced between patients randomised to the omalizumab and placebo arms (Table 1); for the PK/PD population, the baseline total/free IgE levels were similar in both treatment arms in the PK/PD population (Table 1).

**Table 1:** Demographic and baseline patient characteristics.

Full analysis set (FAS)	Omalizumab N = 306	Placebo N = 302	Total N = 608
Mean age, years ± SD	45.8 ± 12.1	47.1 ± 11.6	46.5 ± 11.9
Male, n (%)	138 (45.1)	142 (47.0)	280 (46.1)
Mean bodyweight, kg ± SD	62.3 ± 11.1	62.8 ± 10.3	62.5 ± 10.7
Total IgE, IU/mL ± SD^a^	270.9 ± 180.5	280.6 ± 176.4	275.7 ± 178.4
Duration of asthma, year ± SD	14.2 ± 12.8	15.3 ± 13.6	14.7 ± 13.2
Baseline AQLQ score, mean ± SD	4.4 ± 1.0	4.6 ± 1.0	4.5 ± 1.0
Baseline ACQ score, mean ± SD	1.7 ± 0.6	1.6 ± 0.6	1.7 ± 0.6
Predicted FEV_1_%, mean ± SD	63.5 ± 12.0	63.0 ± 12.7	63.3 ± 12.4
Blood EOS level, cells/μL ± SD	296.4 ± 258.9	283.4 ± 223.9	290.0 ± 242.1
Severe asthma^b^, n (%)	145 (47.4)	143 (47.4)	288 (47.4)
Rescue medication use (RMU, daily number of puffs), mean ± SD	1.8 ± 2.0	2.1 ± 2.9	1.9 ± 2.5
Patients who performed RAST/ImmunoCAP, n (%)	303 (99.0)	297 (98.3)	600 (98.7)
Patients with skin prick test record, n (%)	183 (59.8)	166 (55.0)	349 (57.4)
**Baseline allergen profile**^c^			
With PAR, n (%)	71 (23.2)	86 (28.5)	157 (25.8)
Skin allergy *Dermatophagoides farinae* positive, n (%)	131 (73.2)	107 (65.6)	238 (69.6)
Skin allergy *D. pteronyssinus* positive, n (%)	127 (70.9)	106 (65.0)	233 (68.1)
Skin allergy cockroach positive, n (%)	48 (26.8)	36 (22.1)	84 (24.6)
Skin allergy dog dander positive, n (%)	43 (24.0)	39 (23.9)	82 (24.0)
PK/PD set	Omalizumab n = 60	Placebo n = 64	Total n = 124
Baseline free/total IgE, IU/mL ± SD	252.0 ± 174.3	283.5 ± 174.9	268.3 ± 174.6

ACQ, Asthma Control Questionnaire; AQLQ, Asthma Quality of Life Questionnaire; EOS, eosinophil; FAS, full analysis set; FEV_1_, forced expiratory volume in 1 s; ICS, inhaled corticosteroid; IgE, immunoglobulin E; N, total number of patients; n, number of patients in subcategories; PAR, perennial allergic rhinitis; PK/PD, pharmacokinetic/pharmacodynamic; RAST, radio-allergosorbent test; RMU, rescue medication use; SD, standard deviation.

a For IgE measurement 1 UL/mL equates to 2.4 ng/mL.

b Patients' asthma was considered to be severe if they presented with a baseline predicted FEV_1_% of ≤65% plus at least one of the following: baseline ICS dose ≥1000 μg, two night-time awakenings in the 2 weeks before baseline, and baseline ACQ scores ≥1.5.

c Allergen profile was confirmed by records of skin prick test towards the following allergens: *Dermatophagoides pteronyssinus*, *D. farinae*, cat dander, dog dander, cockroaches and mold, or any other perennial or seasonal allergens. The proportion of patients tested positive for each skin allergen was based on total non-missing values (omalizumab, n = 179; placebo, n = 163)

### Serum total IgE level and blood eosinophil count as a predictor of treatment outcome

**Fig. 1:**
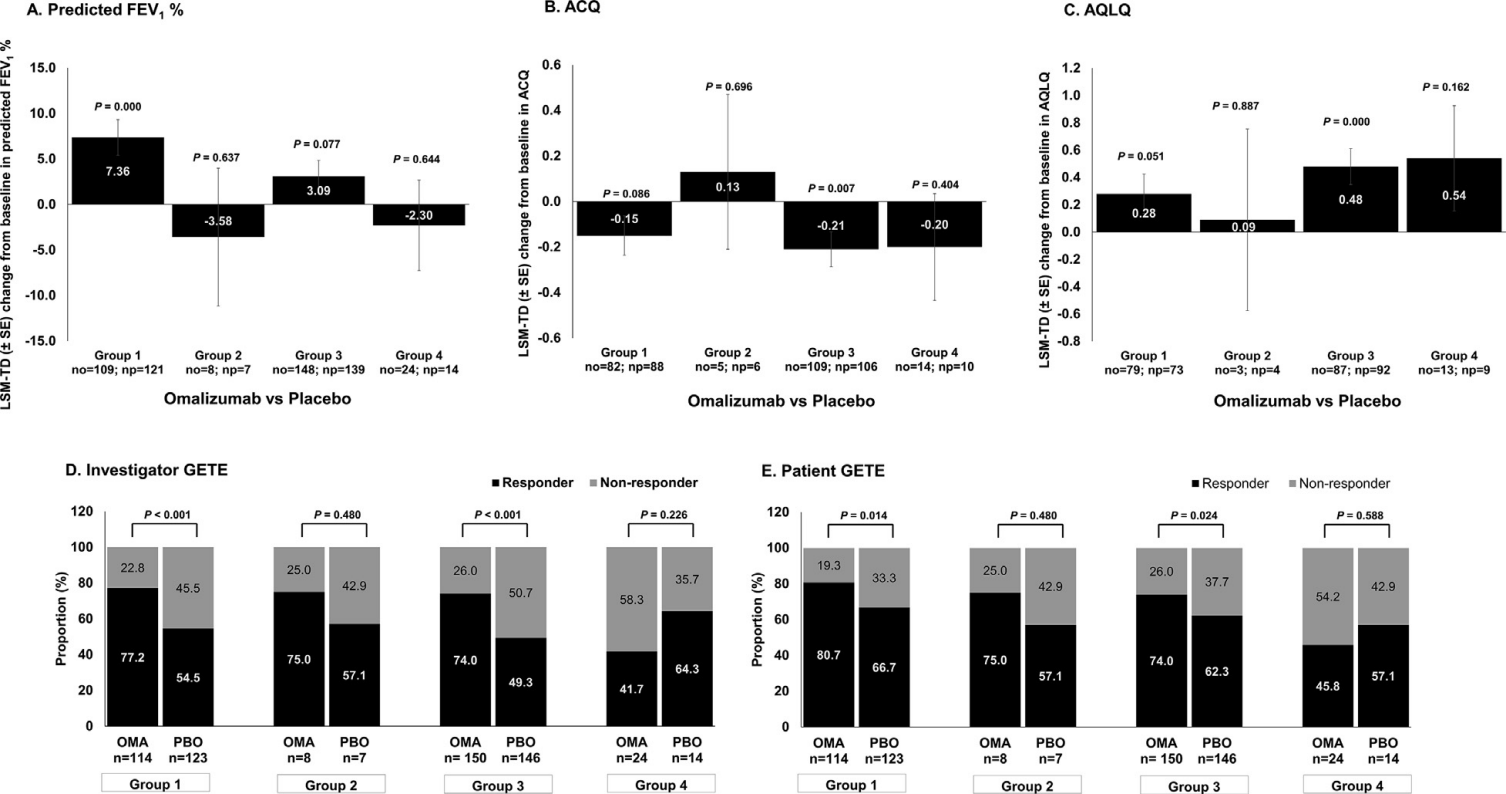
**Treatment outcomes at Week 24 in patients stratified by baseline IgE level and EOS count.** Full Analysis Set. Group 1: EOS ≥300 cells/μL and lgE ≥76 IU/mL; Group 2: EOS ≥300 cells/μL and lgE <76 IU/mL; Group 3: EOS <300 cells/μL and lgE ≥76 IU/mL; Group 4: EOS <300 cells/μL and lgE <76 IU/mL. For IgE measurement 76 UL/mL equates to 182.4 ng/mL. Data were presented as LSM-TD ± SE. LSM-TD indicates treatment difference between omalizumab and placebo groups. P-value presented is for comparison of omalizumab versus placebo. ACQ, Asthma Control Questionnaire; AQLQ, Asthma Quality of Life Questionnaire; CI, confidence interval; EOS, eosinophils; FEV_1_, forced expiratory volume in 1 s; GETE, Global Evaluation of Treatment Effectiveness; LSM, least squares mean; LSM-TD, least squares mean treatment differences; no, number of patients in the omalizumab group; np, number of patients in the placebo group; SE, standard error

Dividing the FAS population into 4 subgroups based on baseline serum total IgE level and blood EOS count, most patients were stratified to Group 1 (high EOS, high IgE) and Group 3 (low EOS, high IgE). The number of patients in the omalizumab and placebo group stratified to Group 2 (high EOS, low IgE) and Group 4 (low EOS, low IgE) was very low, ranging from 3 to 24 patients per group. Omalizumab treatment provided substantial improvement in predicted FEV_1_% from baseline compared with placebo in Group 1 at Week 24 (*P* < 0.001) (Fig. 1A). Compared with placebo, omalizumab treatment also led to substantially greater ACQ score improvement for only Group 3 at Weeks 24 (*P* = 0.007) (Fig. 1B). AQLQ scores showed a trend of improvement in Group 1 (*P* = 0.051), and improvements in Group 3 (*P* < 0.001) in patients treated with omalizumab at Week 24 (Fig. 1C). Based on investigator- and patient-reported GETE assessments, Groups 1 and 3 reported a greater proportion of responders to omalizumab compared with placebo at Week 24 (*P* < 0.05). The responders to omalizumab assessed through the investigator- and patient-reported GETE scores were between 74% and 81% for Groups 1 and 3 (Fig. 1D and E). The results at Week 16 showed a similar trend (see Supplemental Appendix 4).

### Severity of asthma as a predictor of treatment outcome

**Fig. 2:**
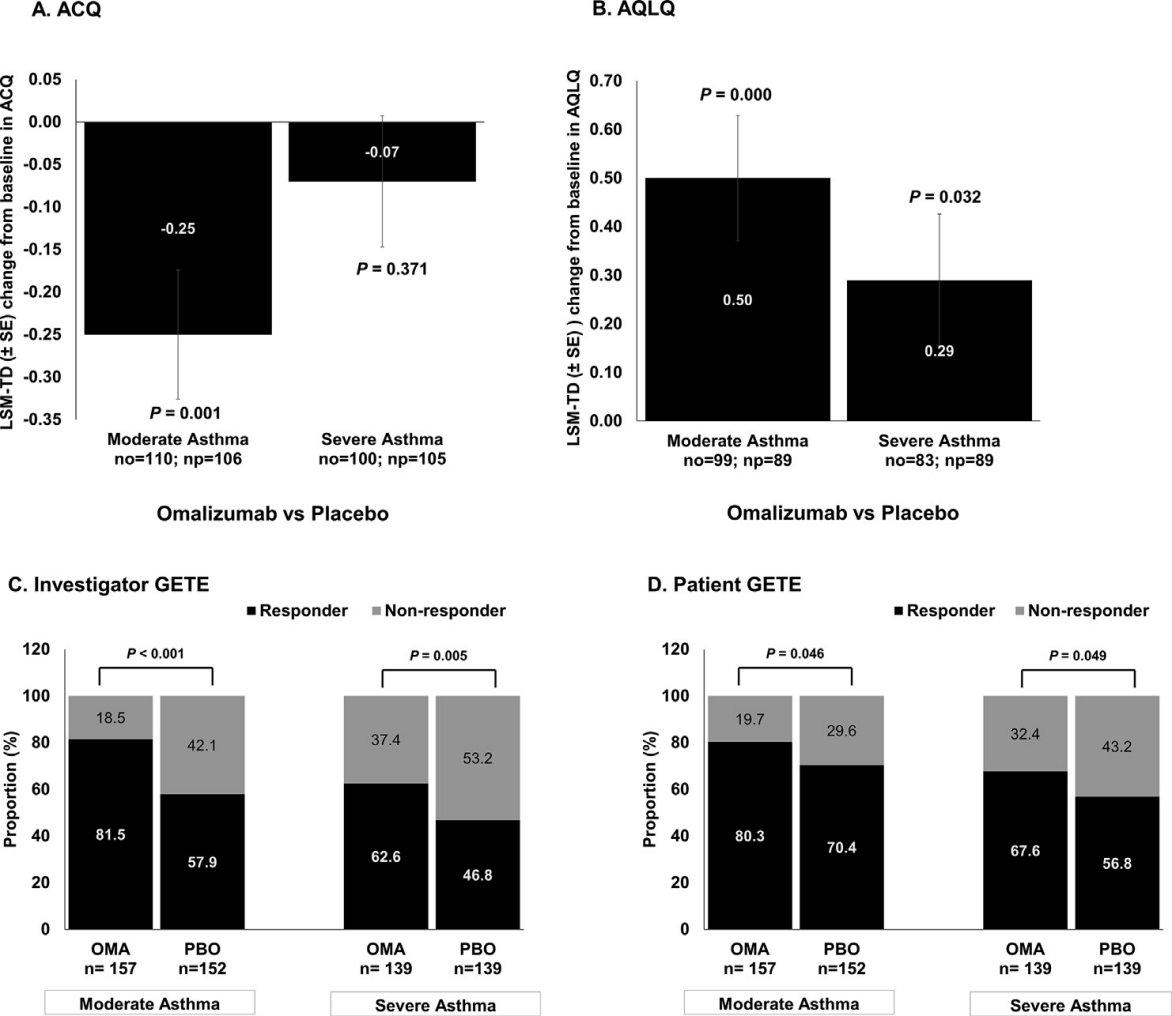
**Treatment outcomes at Week 24 in patients stratified by severity of asthma**. Full Analysis Set. Treatment differences between omalizumab and placebo groups were presented. P-value presented is for omalizumab versus placebo groups. LSM-TD indicates treatment difference between omalizumab and placebo groups. ACQ, Asthma Control Questionnaire; AQLQ, Asthma Quality of Life Questionnaire; Cl, confidence interval; GETE, Global Evaluation of Treatment Effectiveness; LSM, least squares mean; LSM-TD, least squares mean treatment differences; N, total number of patients; n, number of patients; no, number of patients in omalizumab group; np, number of patients in placebo group; SE, standard error

At Week 24, omalizumab treatment substantially improved ACQ scores from baseline compared with placebo in moderate asthmatic patients (*P* = 0.001) (Fig. 2A). Omalizumab treatment also led to a greater improvement in AQLQ scores from baseline to Week 24 in both moderate asthmatic (*P* < 0.001) and severe asthmatic (*P* = 0.032) patient (Fig. 2B). Based on the GETE assessments from both investigators andpatients, a greater proportion of both moderate and severe asthmatic patients generally responded to omalizumab compared with placebo (*P* < 0.05) (Fig. 2C and D). The results at Week 16 showed a similar trend (see Supplemental Appendix 5). We observed a good concordance between investigator and patient assessments at both Weeks 16 and 24.

### Allergen profiles as a predictor of treatment outcome

**Fig. 3:**
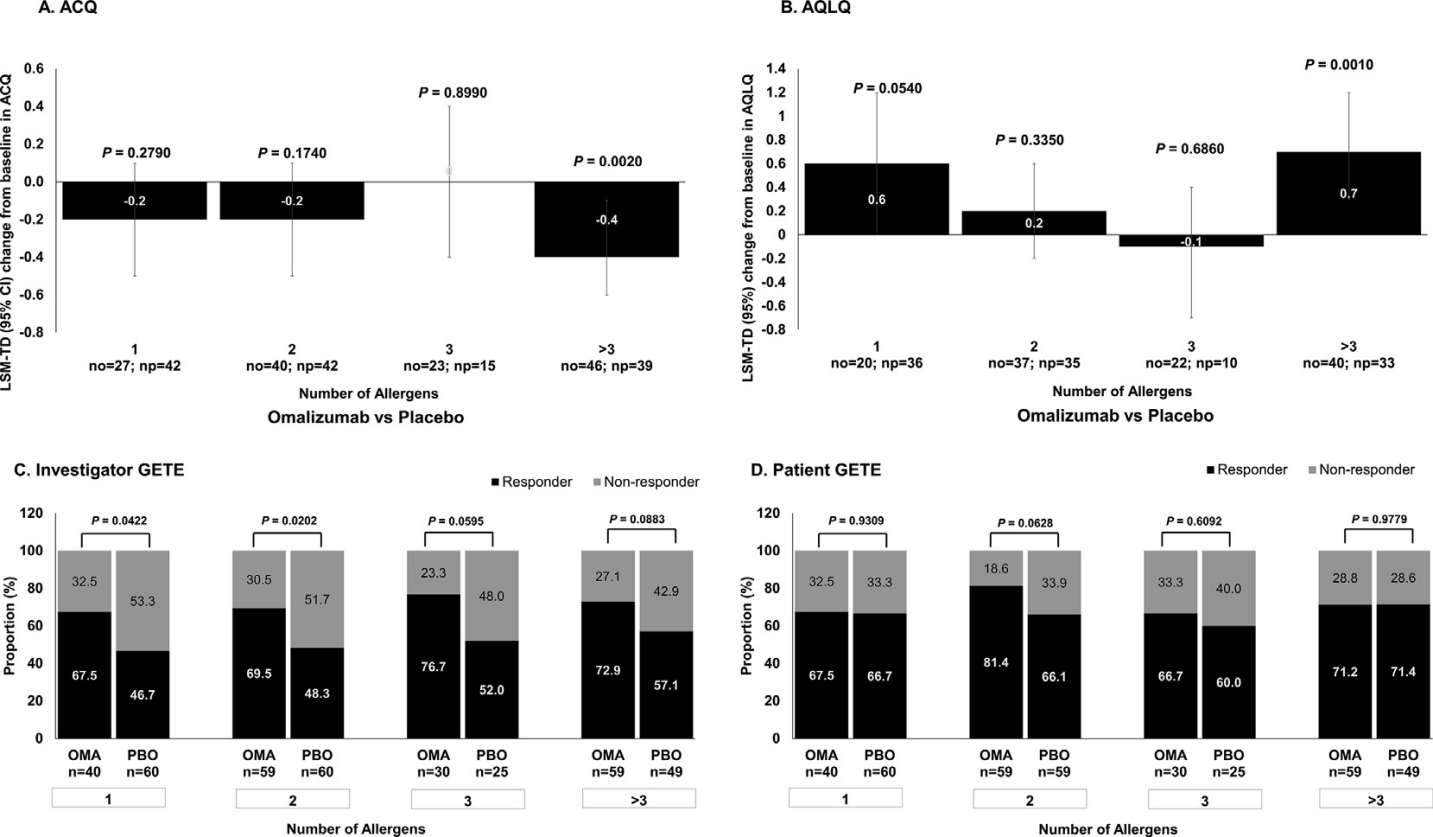
**Efficacy outcomes with omalizumab and placebo at Week 24 in patients categorized by number of sensitized allergens.** Full Analysis Set. P-value is for omalizumab versus placebo.LSM-TD indicates treatment difference between omalizumab and placebo groups. ACQ, Asthma Control Questionnaire; AQLQ, Asthma Quality of Life Questionnaire; CI, confidence interval; GETE, Global Evaluation of Treatment Effectiveness; LSM, least squares mean; LSM-TD, least squares mean treatment differences; N, total number of patients; n, number of patients; no, number of patients in the omalizumab group; np, number of patients in the placebo group; OMA, omalizumab; PAR, perennial allergic rhinitis; PBO, placebo; SE, standard error

**Fig. 4:**
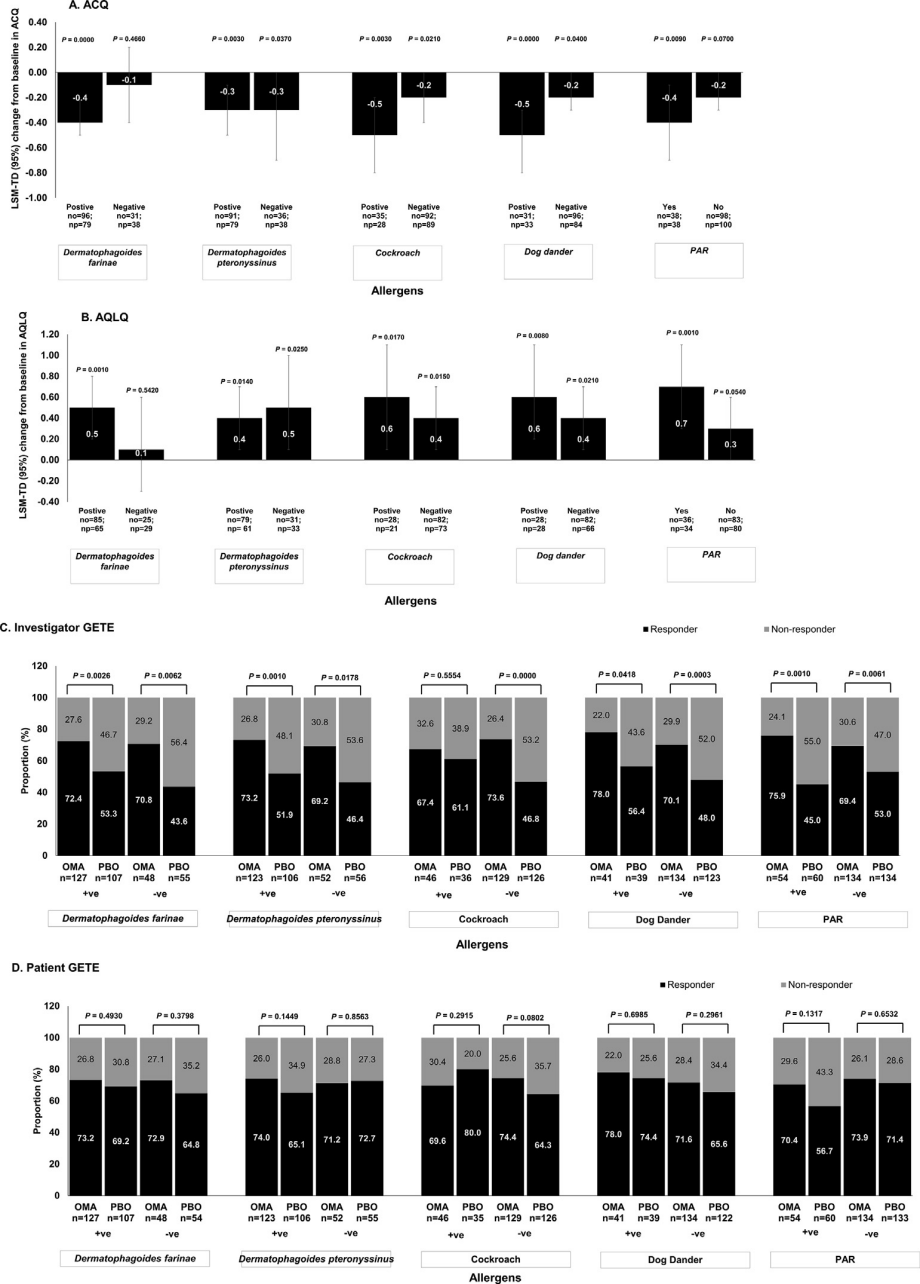
**Efficacy outcomes with omalizumab and placebo at Week 24 in patients categorized by allergen type and PAR.** Full Analysis Set. Most common allergens reported in this study population were presented. P-value is for omalizumab versus placebo. LSM-TD indicates treatment difference between omalizumab and placebo groups. ACQ, Asthma Control Questionnaire; AQLQ, Asthma Quality of Life Questionnaire; CI, confidence interval; GETE, Global Evaluation of Treatment Effectiveness; LSM, least squares mean; LSM-TD, least squares mean treatment differences. N, total number of patients; n, number of patients; no, number of patients in the omalizumab group; np, number of patients in the placebo group; OMA, omalizumab; PAR, perennial allergic rhinitis; PBO, placebo; SE, standard error

Improvements in ACQ scores were observed at Week 24 in patients treated with omalizumab compared with placebo in patients sensitised to >3 allergens (*P* = 0.002) (Fig. 3A), those with a positive skin reaction to *Dermatophagoides farinae* (*P* < 0.001), *D. pteronyssinus* (*P* = 0.003), cockroach (*P* = 0.003), and dog dander (*P* < 0.001), as well as a history of PAR (*P* = 0.009). However, improvements in ACQ scores were also seen in patients non-sensitised to *D. pteronyssinus* (*P* = 0.037), cockroach (*P* = 0.021), and dog dander (*P* = 0.040) (Fig. 4A). Greater improvement in AQLQ scores at Week 24 in omalizumab-treated patients compared with placebo was observed in patients sensitised to >3 allergens (*P* = 0.001) (Fig. 3B), those who had a positive skin reaction to *D. farinae* (*P* = 0.001), *D. pteronyssinus* (*P* = 0.014), cockroach (*P* = 0.017), and dog dander (*P* = 0.008), as well as a history of PAR (*P* = 0.001). However, improvement in AQLQ scores were also seen in patients non-sensitised to *D. pteronyssinus* (*P* = 0.025), cockroach (*P* = 0.015), and dog dander (*P* = 0.021) (Fig. 4B). Thus, the greater effects of omalizumab over placebo were consistent in patients sensitised to >3 antigens and with a history of PAR. There were more responders to omalizumab treatment compared with placebo as assessed by both the investigator- and patient-reported GETE scores, regardless of the number or type of allergen (Figs. 3C–D and 4C–D) (Besides Patient-GETE for patients sensitised to >3 allergens, and for patients not sensitised to *D. pteronyssinus*). The baseline IgE levels of each subgroup were analysed. Baseline IgE levels did not differ substantially between the omalizumab and placebo groups for all allergen profile subgroups *(P* > 0.05) (Supplementary Appendix 6).

### Free IgE suppression as a predictor of treatment outcome

Supplemental Appendix 7 shows that the total IgE level in the placebo group remained stable from baseline to Week 24, while the total IgE level in the omalizumab group increased from baseline to Week 24 as expected. The free IgE values from baseline to Week 24 exceeded the ULOQ of 150 ng/mL for the majority of patients in the placebo group. Thus, an accurate estimation could not be obtained, but indicated that free IgE reduction was not observed. The omalizumab group demonstrated that suppression of free IgE to the level of <50 ng/mL (20.83 IU/mL) was achieved at both Weeks 1 and 24 (Supplemental Appendix 7).

**Fig. 5:**
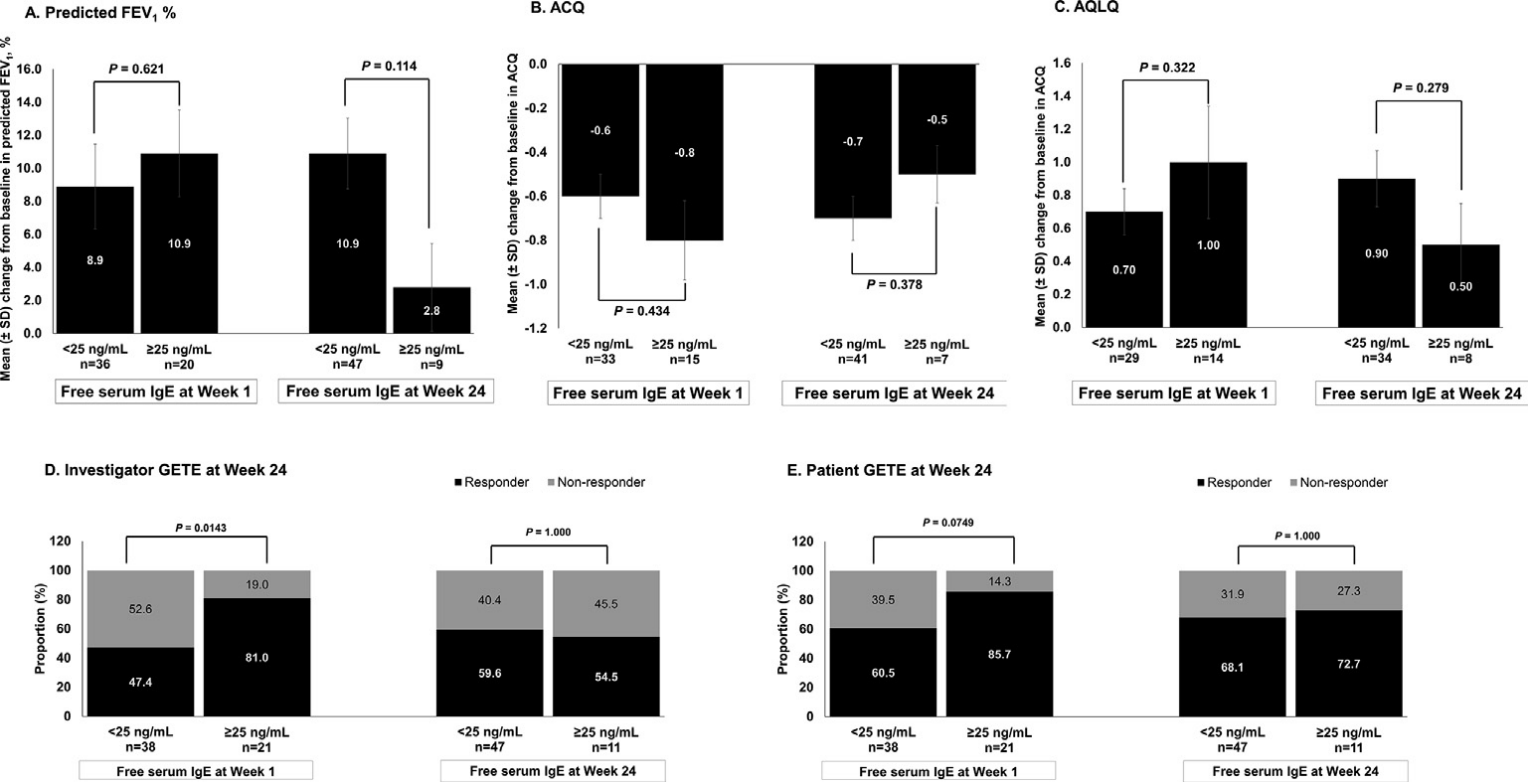
**Efficacy outcomes with omalizumab and placebo at Week 24 in patients categorized by free serum IgE levels**. PK/PD population. Data presented as mean ± SD, except for GETE scores, where the data are presented as percentage of patients in responders versus non-responders. For IgE measurement 25 ng/mL equates 10.42 IU/mL. *P*-value is for free serum IgE level <25 ng/mL group versus ≥25 ng/mL group. ACQ, Asthma Control Questionnaire; AQLQ, Asthma Quality of Life Questionnaire; CI, confidence interval; FEV _1_, forced expiratory volume in 1 s; GETE, Global Evaluation of Treatment Effectiveness, IgE, immunoglobulin E; N, total number of patients; n, number of patients; NR, non-responders; PK/ PD, pharmacokinetic/pharmacodynamic; R, responders; SD, standard deviation

There were no compelling differences in treatment responses in terms of predicted FEV_1_%, AQLQ score, and ACQ score at Week 24 between patients with free IgE <25 ng/mL and ≥25 ng/mL (<10.42 or >10.42 IU/mL) at both Weeks 1 and 24 (*P* > 0.05). Investigator- and patient-reported GETE scores showed that approximately 47%–86% of patients from each subgroup were responders to omalizumab at Week 24 (Fig. 5).

## Discussion

It was previously demonstrated that clinical efficacy and safety of omalizumab were comparable among Chinese and Caucasian patients with moderate-to-severe asthma, supporting therapeutic effectiveness irrespective of race, ethnicity, and geographical factors.^30^^,^^31^ This study is the first to examine the predictors for treatment efficacy of omalizumab over placebo in a Chinese population with moderate-to-severe persistent allergic asthma. We found that baseline IgE level and allergen profile (number/history of PAR) are potential predictors of treatment response to omalizumab, while asthma severity and baseline eosinophil count were not associated with treatment response to omalizumab. We found that all patients treated with omalizumab achieved levels of circulating free IgE under 50 ng/mL during the treatment period (1 week and 24 weeks). Similar clinical outcomes were observed in the subset of patients who achieved free IgE levels of <25 and ≥ 25 ng/mL. Our current findings were consistent with previous similar studies that were conducted in a patient population of other ethnicities, and will provide reference to clinical practice for the use of omalizumab in treating moderate-to-severe persistent allergic asthma in Chinese patients in East Asia and Asia Pacific, as well as in countries and other regions with multiple ethnicities.

We examined the predictor values of the combination of baseline serum total IgE and blood EOS level for treatment efficacy of omalizumab over placebo. It should be emphasised that with the low number of patients with serum total IgE <76 IU/mL in this study, thus majority of the patients were clustered into Groups 1 and 3. Notably, patients with baseline serum total lgE ≥76 IU/mL, regardless of baseline blood EOS level, were generally associated with better efficacy response to omalizumab. The finding that patients with baseline serum total lgE ≥76 IU/mL benefitted from omalizumab compared to placebo was consistent with previous studies. Bousquet et al (2007) showed that baseline total IgE (≥76 IU/mL) was the only predictor of omalizumab's efficacy (asthma exacerbation, AQLQ, physician's overall assessment) in the INNOVATE study.^14^ However, the pooled analysis, which included 7 randomised studies, demonstrated treatment benefits of omalizumab over placebo irrespective of baseline IgE levels.^14^ Abdelaty (2012) demonstrated that responders to omalizumab had a higher mean baseline IgE (400 IU/mL in responders vs 320 IU/mL in non-responders).^15^ Kallieri et al (2017)^16^ showed that patients with higher mean baseline serum IgE levels benefited from prolonged treatment (32 weeks) with omalizumab (704 IU/mL in responder vs 121 IU/mL in non-responder). In our study, there is no consistent observation that baseline blood EOS count <300 or ≥300 cells/μL was associated with the efficacy of omalizumab. Previous studies have shown that when asthma exacerbation was assessed as treatment response, patients with higher baseline EOS levels had a greater response to omalizumab. A *post-hoc* analysis of 2 clinical studies showed greater reductions in exacerbation rate with omalizumab in patients with higher (≥300 cells/μL) versus lower (<300 cells/μL) EOS count.^32^ The *post-hoc* analysis of the EXTRA study showed a greater reduction in exacerbation rate in a patient subgroup with high (≥260 cells/μL at baseline) versus low (<260 cells/μL at baseline) EOS count during the 48-week omalizumab treatment period.^18^ When GETE was assessed as the treatment response, the EOS level was not observed to be an efficacy predictor for omalizumab versus placebo. Kallieri et al. (2017) showed that EOS counts were not significantly different from responders and non-responders to omalizumab at Week 16, yet late responders (responded at Week 32) had higher blood EOS.^16^ Humbert et al (2018) showed that the effectiveness of omalizumab was similar in those with high (≥300 cells/μL) and low (<300 cells/μL) EOS counts at 4–6 months after treatment.^33^ Consistently, we showed that GETE scores were not different between groups with high or low EOS counts at Weeks 16 and 24. This is consistent with the observations in the early responders (Week 16) in the study of Kallieri et al. (2017),^16^ and in the real-world study by Humbert et al (2018).^24^ However, it should be noted that prescriptions of omalizumab are based on a dosing table accounting for total serum IgE and the bodyweight of the patient, which aimed to achieve an average serum free IgE of 25 ng/mL (with 95% of patients below 50 ng/mL), a level associated with clinical improvement.^19^ Doubts remain, therefore, if baseline IgE levels could be a valuable predictor of omalizumab efficacy, and what would be the most appropriate cut-off level for such an assessment. Nevertheless, it is suggested that allergic status such as skin positive response and allergen specific IgE should be taken into considerations to prescript omalizumab when serum total IgE<76 IU/mL.

Most studies investigating predictor factors for omalizumab treatment responses were conducted in patients with severe persistent allergic asthma^15^, ^16^, ^17^^,^^29^ with very few reported for moderate allergic asthma patients.^32^ The findings reported here are consistent with previous studies conducted on global populations, wherein Chinese patients with both moderate and severe allergic asthma showed similar responses to treatment with omalizumab compared with placebo.

It was also demonstrated that patients who showed improvement in asthma control and quality of life (QoL) with omalizumab compared with placebo were those with the highest allergic burden. These findings are consistent with a previous study, which demonstrated that responders to omalizumab were sensitised to a higher number of allergens.^15^ House dust mites are a major perennial allergen source and a significant cause of allergic rhinitis and allergic asthma.^29^ Our finding is consistent with a previous investigation, which demonstrated that dust mites, cockroaches, and dog dander were amongst the most common inhaled sensitizing allergens involved in the manifestation of allergic asthma in China.^34^ The current study also demonstrated that a history of PAR was associated with improved QoL with omalizumab treatment compared with placebo. This finding is consistent with the SOLAR study, which showed that, in addition to reducing asthma exacerbation, omalizumab also improved asthma and rhinitis QoL scores, and led to significant improvement in rhinitis symptoms in patients with concomitant asthma and PAR.^35^ Previous studies have demonstrated that omalizumab significantly reduced symptom severity and rescue-antihistamine use, as well as significantly improving rhinitis or rhino-conjunctivitis-related QoL in patients with seasonal allergic rhinitis (SAR) or PAR.^35^, ^36^, ^37^ Improvement in the QoL of patients with allergic asthma treated with omalizumab may be attributed to concurrent improvement of PAR symptoms. In addition, we showed that baseline IgE levels did not differ substantially between omalizumab and placebo group for all the allergen profile subgroups, indicating that the differences in baseline IgE was not the driving force for the response to omalizumab.

In the current study, patients treated with omalizumab had the expected increase in total IgE from baseline to Weeks 1 and 24, which is due to the build-up of the immunoglobulin G (IgG)–IgE complex that has a slower clearance compared with free IgE.^38^ Consistent with a previous study,^39^ both the serum total and free IgE remained fairly stable in the placebo group from baseline up to the end of study. Previous studies have shown that monitoring free IgE serum concentrations in patients treated with omalizumab does not predict clinical response.^40^, ^41^ We showed that free-IgE reduction to levels of <25 ng/mL at both Weeks 1 and 24 was not associated with better omalizumab treatment response. Thus, the analysis indicated that Chinese patients who were treated according to the omalizumab dosing table could achieve adequate free-IgE reduction (<50 ng/mL), and free-IgE reduction to levels of <25 ng/mL at both Weeks 1 and 24 do not serve as the predictor for better omalizumab treatment response.

Limitation of the current analysis includes those inherent to *post-hoc* analyses, and the small sample size of the PK/PD population. For allergen profile testing, we have only conducted skin prick tests for 6 groups (13 kinds) of selected common allergens, and included the results for few most common allergens reported in this study population. Another limitation of the current study was that the study was not designed for long-term outcomes, thus we only reported results up to 24 weeks. However, previous studies have shown strong evidence that omalizumab can provide long-term efficacy in management of allergic asthma.^42^^,^^43^

## Conclusion

In Chinese patients with moderate-to-severe allergic asthma, baseline total IgE and allergen profile (number/history of PAR) could potentially serve as the predictor of treatment response to omalizumab. The findings from this *post-hoc* analysis warrant further verification in future large prospective studies.

## Ethics approval and consent to participate

The study was conducted in accordance with the current Good Clinical Practice, and the protocol was approved by Independent Ethics Committee or Institutional Review Board for each centre. Written informed consent was obtained from each enrolled patient.

## Data availability statement

The data that support the findings of this study are available from the corresponding author upon reasonable request.

## Consent for publication

All authors agreed to the publication of this work.

## Funding statement

The clinical study was funded by Novartis Pharma AG, Basel, Switzerland. This *post-hoc* analysis was sponsored by Beijing Novartis Pharma, China and “Precision medicine research program” of the 10.13039/501100012166National key research and development project of China, China (2016YFC0905800). Beijing Novartis Pharma, China, funded the medical writing and editorial assistance for this article.

## Author contributions

JL, NSZ, and LW led the study conception and design, data analyses and interpretation. JL, NSZ, LW, RF and JY led the manuscript writing. All authors contributed in study conception and design, data analyses and interpretation, writing the manuscript and critical revision of drafts. All authors read and approved the final manuscript.

## Declaration of competing interest

L. Wang, R. Fogel, X. Jaumont, and J. Yang are employees of Novartis. R. Fogel owns stock in Novartis.

J. Li, C. Wang, C. Liu, J. Kang, L. Kong, Y. Huang, S. Liu, M. Huang, and N. Zhong have no competing interest to disclose.
