# Supplementary material for: Efficacy predictors of omalizumab in Chinese patients with moderate-to-severe allergic asthma: Findings from a post-hoc analysis of a randomised phase III study
Source: World Allergy Organ J. 2020 Nov 24;13(12):100469. doi: 10.1016/j.waojou.2020.100469 (PMC8461112; doi:10.1016/j.waojou.2020.100469)
Supplement: Multimedia component 1 [file mmc1.docx]

**Supplementary Information:**

**Supplemental Appendix 1: List of 42 investigators in China Omalizumab Study Group**

| Li Jing | The 1st Affiliated Hospital of Guang Zhou Medical University |
| --- | --- |
| Wang Changzheng | Xinqiao Hospital, Third Military Medical University |
| Bai Chunxue | Shanghai Zhongshan Hospital, Fudan University |
| Liu Shuang | Beijing Anzhen Hospital |
| Wang Guangfa | Peking University First Hospital |
| Yan Xixi | The 2nd Hospital of Hebei Medical University |
| Chen Ping | The General Hospital of Shenyang Military Command |
| Wu Changgui | Xijing Hospital, Fourth Military Medical University |
| Yang Heping | Southwest Hospital, Third Military Medical University |
| Cui Shehuai | Daping Hospital, Third Military Medical University |
| Shen Ce | Shanghai 6th Hospital |
| Li Huiping | Shanghai Pulmonary Hospital |
| Zhang Suiyang | General Hospital of 2nd Artillery PLA |
| Chen Hangwei | The Military General Hospital of Beijing |
| Xia Guoguang | Beijing Jishuitan Hospital |
| Sun Tieying | Beijing Hospital |
| Wang Haoyan | Beijing Friendship Hospital |
| Liu Chuntao | West China Hospital, Sichuan University |
| Kang Jian | The 1st Hospital Affiliated to China Medical University |
| Sun Xiuzhen | The 2nd Affiliated Hospital of Xi'an Jiaotong University |
| Wen Zhongguan | The 2nd Affiliated Hospital, General Hospital of PLA |
| Lin Jiangtao | China-Japan Friendship Hospital |
| Shi Minhua | The 2nd Affiliated Hospital, Suzhou Medical University |
| Huang Jiannan | The 1st Affiliated Hospital of Suzhou University |
| Zhou Jianying | The 1st Affiliated Hospital of ZheJiang University College |
| Jin Faguang | Tangdu Hospital, Fourth Military Medical University |
| Hu Chengping | The 1st Xiangya Hospital of Central South University |
| Chen Ping | The 2nd Xiangya Hospital of Central South University |
| Sun Shenghua | The 3rd Xiangya hospital of Central South University |
| Xin Jianbao | Wuhan Union Hospital Affiliated to Tongji Medical University |
| Cai Shaoxi | Nanfang Hospital, Affiliated to Southern Medical University |
| Xie Canmao | The First Affiliated Hopsital, SunYat-Sen University |
| Chen Zhengxian | People’s Hospital of Guangdong Province |
| Li Qiang | Shanghai Changhai Hospital, Second Military Medical University |
| Shi Yi | Nanjing General Hospital of Nanjing Military PLA |
| Huang Mao | People's Hospital of Jiangsu Province |
| Wan Huanying | Ruijin Hospital Affiliated to Shanghai Jiao Tong University |
| Huang Yijiang | Hainan Province People's Hospital |
| Zhang Jie | The 2nd Hospital of Jilin University |
| Kuang Jiulong | The 2nd Affiliated Hospital of Nanchang University |
| Liu Guanghui | Wuhan Tongji Hospital Affiliated to Tongji Medical University |
| Zhong Xiaoning | The 1st Affiliated Hospital of Guangxi Medical University |

**Supplemental Appendix 2: Inclusion and Exclusion Criteria**

**Inclusion criteria**

Patients who met the following criteria at the time of screening (Visit 1) and Visit 2 were eligible for inclusion in this study:

1. Written informed consent was obtained before any assessment was performed, including any adjustments to medication during the screening period

2. Age 18–75 years inclusive

3. Serum baseline total IgE level ≥30 to ≤700 IU/mL and bodyweight >20 kg and ≤150 kg. Patients with a total IgE level of ≤76 IU/mL required an unequivocal positive RAST or ImmunoCAP test to be eligible

4. Confirmed diagnosis of asthma for a duration of ≥1 year at screening, and a history of asthma that was not adequately controlled with GINA (2009) Step-4 therapy

5. Receiving medium-to-high dose inhaled corticosteroid >500 μg beclomethasone (BDP), or equivalent, plus regularly inhaled long-acting β agonist, either separately or in combination, for at least 8 weeks prior to screening

6. Meet one of the following asthma exacerbations eligibility criteria prior to the screening period. All exacerbations required the use of additional systemic steroids and/or intravenous theophylline (aminophylline) to qualify:

• Had at least 2 reported exacerbations in the previous 12 months OR

• Three reported exacerbations in the previous 24 months; 1 of these exacerbations had to have occurred in the previous 12 months OR

• Had been admitted to hospital as an in-patient (including intensive care unit) or received urgent care as an out-patient (including emergency room or observational room treatment) in the past 12 months for an asthma exacerbation

7. During any 1 week of the 4-week stable dose run-in period (immediately prior to randomization), patients exhibited inadequate symptom control as demonstrated by one or more of the following (in keeping with GINA [2009] guidelines):

• Daytime symptoms more than twice per week (i.e. ≥3 times in a 7-day period)

• Any limitation on activity

• Any nocturnal symptoms or awakenings

• Need for reliever/rescue treatment more than twice per week (i.e. ≥3 times in a 7-day period)

• High variance in daily PEF (mean of the daily variance over a 1-week period was ≥20%)

8. Positive reaction to at least 1 perennial aeroallergen (e.g. dog, cat, cockroach [whole body], dust mite [*Dermatophagoides farinae, D. pteronyssinus*]) as documented by a historical skin prick test within 12 months prior to screening, or at Visit 1. Alternatively, if no positive skin prick test was available, or there was no historical record of reaction to cockroach, then a positive RAST or ImmunoCAP test to at least one relevant perennial aeroallergen was required at screening

9. Demonstrated ≥12% (and 200 mL) increase in FEV_1_ within 30 minutes after taking salbutamol/albuterol. If during the Visit 1 reversibility assessment, change in FEV_1_ was ≥8% and <12%, then the patient was considered as ‘suitable for re-assessment’and could repeat the assessment at Visit 2

10. FEV_1_ ≥40% and <80% of the predicted normal value for the patient (using local standards), after withholding bronchodilators) at Visit 2

11. Compliance with completion of PEF/eDiary during the run-in period – compliance was defined as ≥85% of the PEF assessments and ≥85% of the morning or evening eDiary sessions completed correctly in the 28 days prior to randomization. At the investigators’ discretion, the run-in period could be extended to ensure that at least 85% of the PEF/eDiary data was collected over a 28-day period

**Exclusion criteria**

Patients fulfilling any of the following criteria at the time of screening (Visit 1), unless listed otherwise, were not eligible for inclusion in this study:

1. Use of other investigational drugs at the time of enrollment, or within 30 days or 5 half-lives of enrollment, whichever was longer. For biological agent-based investigational drugs, such as monoclonal antibodies, at least 6 months needed to have passed between the last administration of the drug and the patient’s screening visit

2. Known hypersensitivity to any ingredients, including excipients (sucrose, histidine, polysorbate 20) of the study medication or drugs related to omalizumab (e.g. monoclonal antibodies, polyclonal gamma globulin)

3. History of malignancy of any organ system (other than localized basal cell carcinoma of the skin), treated or untreated, regardless of whether there was evidence of local recurrence or metastases

4. A history of food- or drug-related severe anaphylaxis

5. Active lung disease other than allergic asthma (e.g. chronic bronchitis, COPD)

6. A clinically significant abnormality on a 12-lead ECG recorded at Visit 1

7. A clinically significant abnormality on a chest x-ray at screening

8. Elevated serum IgE levels for reasons other than allergy (e.g. parasite infections, hyper-immunoglobulin E syndrome, Wiskott-Aldrich syndrome, or bronchopulmonary aspergillosis)

9. Patients with significant underlying medical conditions that could affect the interpretation of results (e.g. infection, hematological disease, renal, hepatic, coronary heart disease or other cardiovascular disease, cerebrovascular disease, endocrinologic or gastrointestinal disease) within the previous 12 months

10. A lower respiratory tract infection, an upper respiratory tract infection, or having had an asthma exacerbation episode that was ongoing or had ended within 1 month of Visit 1

11. With clinically significant laboratory abnormalities (not associated with the study indication) at Visit 1

12. Currently smoke, or a former smoker with a smoking history of >10 pack-years (defined as the number of packs of 20 cigarettes smoked per day multiplied by number of years the patient smoked). A former smoker had abstained for a minimum of 12 months before randomization

13. Pregnant or nursing (lactating) women or women of child-bearing potential not using acceptable birth control measures

14. Those receiving prohibited medication or who had previously received omalizumab

15. Anyone considered potentially unreliable or where it was envisaged the patient might not consistently attend scheduled study visits

16. Those with a history of drug or alcohol abuse

17. Anyone unable or unwilling to comply with study procedures and visits (e.g. electronic peak flow measurements, spirometry, blood draws, eDiary or questionnaires). This included patients whose sleep-wake pattern prevented them from completing the electronic peak flow measurements and eDiary assessments in a consistent and regular manner.

**Supplemental Appendix 3: Subgroup analyses**

**Subgroup analysis 1: Baseline blood EOS counts and total IgE level**

The FAS population was divided to 4 subgroups based on baseline blood EOS and IgE level:

Group 1–EOS ≥300 cells/μL and IgE ≥76 IU/mL

Group 2–EOS ≥300 cells/μL and IgE <76 IU/mL

Group 3–EOS <300 cells/μL and IgE ≥76 IU/mL

Group 4–EOS <300 cells/μL and IgE <76 IU/mL

Predicted FEV_1_ %, ACQ score, AQLQ score, and GETE scores were assessed in these patients at Weeks 16 and 24.

**Subgroup analysis 2: Asthma severity at baseline**

FAS patients were categorized as having moderate or severe asthma based on the following criteria:

Severe asthma – patients presented with a predicted baseline FEV_1_ % of ≤65% plus at least one of the following: baseline ICS dose ≥1000 μg, 2 night-time awakenings in the 2 weeks before baseline, and baseline ACQ scores ≥1.5.

Moderate asthma – patients not meeting the criteria for severe asthma were considered to have moderate asthma.

ACQ score, AQLQ score, and GETE scores were assessed in these patients at Weeks 16 and 24

**Subgroup analysis 3: Allergen profiles (allergen number, type, history of PAR)**

FAS patients were stratified based on:

The number of allergens that patients experienced, allergen number (1, 2, 3, and ≥3) based on history of food/drug allergy, history of seasonal allergy, and positive skin prick tests (allergens tested includes *Dermatophagoides pteronyssinus*, *D. farinae*, cat dander, dog dander, cockroaches, mix pollens and mix molds).

History of perennial allergic rhinitis

Type of allergens

Skin allergy – dog dander positive/negative

Skin allergy – cockroach positive/negative

Skin allergy – *D. farinae* positive/negative

Skin allergy –*D. pteronyssinus* positive/negative

Skin allergy – pollen positive/negative

Skin allergy – mold positive/negative

ACQ score, AQLQ score, and GETE scores were assessed at Week 24.

**Subgroup analysis 4: Free IgE level at Day 8 and Week 24**

PK/PD patients (omalizumab treatment group) with data on serum free IgE and
Week 24 were stratified based on:

Serum free IgE level at Day 8 (<25 ƞg/mL and ≥25 ƞg/mL)

Serum free IgE level at Week 24 (<25 ƞg/mL and ≥25 ƞg/mL)

Predicted FEV_1_ %, ACQ score, AQLQ score, and GETE scores were assessed at
Week 24.

**Supplemental Appendix 4: Treatment outcomes at Week 16 in patients stratified by baseline IgE level and EOS count**

| **Parameter** | | **Group 1 EOS ≥300 cells/µLand lgE ≥76 IU/mL** | **Group 2**  **EOS ≥300 cells/µL and lgE <76 IU/mL** | **Group 3 EOS <300 cells/µL and lgE ≥76 IU/mL** | | **Group 4**  **EOS <300 cells/µL and lgE <76 IU/mL** | | |
| --- | --- | --- | --- | --- | --- | --- | --- | --- |
| **LSM-TD change from baseline in predicted FEV_1_ %^a^** | **Mean ± SE** | 5.76±1.990 | −11.81±7.655 | 2.70±1.759 | | −1.06±5.114 | | |
|  | **95% CI**  ***P*-value** | 1.85 to 9.67  0.004 | −26.85 to 3.23  0.124 | −0.75 to 6.16  0.125 | | −11.11 to 8.98  0.835 | | |
|  | **N** | n_o_=109; n_p_=120 | n_o_=8; n_p_=7 | n_o_=147; n_p_=143 | | n_o_=25; n_p_=13 | | |
| **LSM-TD change from baseline in ACQ^a^** | **Mean ± SE** | −0.28±0.084 | 0.47±0.321 | –0.20±0.073 | | –0.12±0.233 | | |
|  | **95% CI**  ***P*-value** | −0.44 to −0.11  0.001 | −0.16 to 1.10  0.145 | −0.34 to –0.05;  0.007 | | −0.57 to 0.34  0.616 | | |
|  | **N** | n_o_=85 ; n_p_=91 | n_o_= 6; n_p_ = 6 | n=118; n_p_=116 | | n_o_=16; n_p_=9 | | |
| **LSM-TD change from baseline in AQLQ^a^** | **Mean ± SE** | 0.17±0.124 | –0.66±0.525 | 0.19±0.111 | | 0.40±0.335 | | |
|  | **95% CI**  ***P*-value** | −0.07 to 0.41  0.169 | −1.69 to 0.37  0.209 | −0.03 to 0.41  0.087 | | −0.26 to 1.06  0.233 | | |
|  | **N** | n_o_=81; n_p_=76 | n_o_=5; n_p_=4 | n_o_=99; n_p_=99 | | n_o_=14; n_p_=9 | | |
| **Investigator-GETE (omalizumab vs placebo)** | | | | | | | | |
| **Responders** | **% (n)** | 80.7 (92) vs 52.0 (64) | 87.5 (7) vs 42.9 (3) | | 71.5 (108) vs 55.1 (81) | | 52.0 (13) vs 64.3 (9) |  |
| **Non-responders** | **% (n)** | 19.3 (22) vs 48.0 (59); | 12.5 (1) vs 57.1 (4) | | 28.5 (43) vs 44.9 (66) | | 48.0 (12) vs 35.7 (5) |  |
|  | ***P*-value** | <0.001 | 0.077 | | 0.004 | | 0.548 |  |
| **Patient-GETE (omalizumab vs placebo)** | | | | | | | | |
| **Responders** | **% (n)** | 78.9 (90) vs 61.0 (75) | 87.5 (7) vs 42.9 (3) | | 68.9 (104) vs 61.9 (91) | | 60.0 (15) vs 71.4 (10) |  |
| **Non-responders** | **% (n)** | 21.1 (24) vs 39.0 (48) | 12.5 (1) vs 57.1 (4) | | 31.1 (47) vs 38.1 (56) | | 40.0 (10) vs 28.6 (4) |  |
|  | ***P*-value** | 0.003 | 0.077 | | 0.226 | | 0.581 |  |

Full Analysis Set.

**^a^**Data were presented as ΔLSM ± SE and 95% CI. ΔLSM indicates treatment difference between omalizumab and placebo groups. *P*-value presented is for comparison of omalizumab versus placebo

For IgE measurement 76 UL/mL equates to 182.4 ng/mL

ACQ, Asthma Control Questionnaire; AQLQ, Asthma Quality of Life Questionnaire; CI, confidence interval; EOS, eosinophils; FEV_1_, forced expiratory volume in 1 second; GETE, Global Evaluation of Treatment Effectiveness; LSM, least squares mean; LSM-TD, least squares mean treatment differences; n_o_, number of patients in the omalizumab group; n_p_, number of patients in the placebo group; SE, standard error

**Supplemental Appendix 5. Treatment outcomes at Week 16 in patients stratified by severity of asthma**

| **Parameter** | | **Week 16** | |
| --- | --- | --- | --- |
|  |  | **Moderate asthma** | **Severe asthma** |
| **LSM-TD change from baseline in ACQ^a^** | **Mean ± SE** | −0.16±0.073 | −0.23±0.074 |
|  | **95% CI**  ***P*-value** | −0.30 to − 0.01  0.032 | −0.38 to −0.08  0.002 |
|  | **N** | n_o_=117; n_p_=114 | n_o_=108; n_p_=109 |
| **LSM-TD change from baseline in AQLQ^a^** | **Mean ± SE** | 0.20±0.111 | 0.18±0.115 |
|  | **95% CI**  ***P*-value** | −0.01 to 0.42  0.067 | −0.05 to 0.40  0.124 |
|  | **N** | n_o_=104; n_p_=95 | n_o_=95;n_p_=93 |

| **Investigator-GETE (omalizumab vs placebo)** |
| --- |

| **Responders** | **% (n)** | 80.5 (128) vs 61.6 (93) | 65.7 (94) vs 46.0 (63) |
| --- | --- | --- | --- |
| **Non-responders** | **% (n)** | 19.5 (31) vs 38.4 (58) | 34.3 (49) vs 54.0 (74) |
|  | ***P*-value** | <0.001 | <0.001 |

| **Patient-GETE (omalizumab vs placebo)** |
| --- |

| **Responders** | **% (n)** | 78.6 (125) vs 68.9 (104) | 64.3 (92) vs 54.7 (75) |
| --- | --- | --- | --- |
| **Non-responders** | **% (n)** | 21.4 (34) vs 31.1 (47) | 35.7 (51) vs 45.3 (62) |
|  | ***P*-value** | 0.051 | 0.109 |

Full Analysis Set.

**^a^**Treatment differences between omalizumab and placebo groups were presented. *P*-value presented is for omalizumab versus placebo groups

**Δ**LSM indicates treatment difference between omalizumab and placebo groups. For GETE scores, data are presented as percentage of patients (number of patients) in omalizumab versus placebo groups

ACQ, Asthma Control Questionnaire; AQLQ, Asthma Quality of Life Questionnaire; Cl, confidence interval; GETE, Global Evaluation of Treatment Effectiveness; LSM, least squares mean; LSM-TD, least squares mean treatment differences; N, total number of patients; n, number of patients; n_o_, number of patients in omalizumab group; n_p_, number of patients in placebo group; SE, standard error

**Supplemental Appendix 6. Baseline IgE of patients with efficacy outcomes at Week 24 in patients categorised by allergen exposure and PAR**

| **Allergen profile** | **Statistics** | **Baseline IgE** | | ***P-*value** |
| --- | --- | --- | --- | --- |
|  |  | **Omalizumab** | **Placebo** |  |
| **ACQ** | | | | |
| **Number of allergens** | |  |  |  |
| **1** | **Number of subject, n (non-missing)** | 27 | 42 |  |
|  | **Mean (SD)** | 291.3 (215.29) | 288.1 (195.61) | 0.9499 |
|  | **Median (Q1─Q3)** | 234.0  (88.0─470.0) | 249.5  (112.0─478.0) |  |
| **2** | **Number of subject, n (non-missing)** | 40 | 42 |  |
|  | **Mean (SD)** | 245.4 (178.05) | 288.4 (171.16) | 0.2681 |
|  | **Median (Q1─Q3)** | 179.5  (111.0─361.0) | 269.0  (134.0─392.0) |  |
| **3** | **Number of subject, n (non-missing)** | 23 | 15 |  |
|  | **Mean (SD)** | 264.2 (174.44) | 247.2 (210.90) | 0.7882 |
|  | **Median (Q1─Q3)** | 184.0  (122.0─428.0) | 144.0  (80.0─423.0) |  |
| **>3** | **Number of subject, n (non-missing)** | 46 | 39 |  |
|  | **Mean (SD)** | 320.6 (184.26) | 344.8 (162.57) | 0.5261 |
|  | **Median (Q1─Q3)** | 305.0  (176.0─443.0) | 344.0  (172.0─480.0) |  |
| ***Dermatophagoides farinae*** | |  |  |  |
| **Positive** | **Number of subject, n (non-missing)** | 96 | 79 |  |
|  | **Mean (SD)** | 285.0 (194.16) | 282.0 (184.16) | 0.9157 |
|  | **Median (Q1─Q3)** | 237.0  (120.5─441.5) | 239.0  (131.0─472.0) |  |
| **Negative** | **Number of subject, n (non-missing)** | 31 | 38 |  |
|  | **Mean (SD)** | 263.9 (162.69) | 313.6 (181.45) | 0.2396 |
|  | **Median (Q1─Q3)** | 220.0  (117.0─376.0) | 292.5  (141.0─455.0) |  |
| ***Dermatophagoides pteronyssinus*** | |  |  |  |
| **Positive** | **Number of subject, n (non-missing)** | 91 | 79 |  |
|  | **Mean (SD)** | 275.6 (186.13) | 301.8 (190.30) | 0.3656 |
|  | **Median (Q1─Q3)** | 231.0  (121.0─386.0) | 247.0  (143.0─478.0) |  |
| **Negative** | **Number of subject, n (non-missing)** | 36 | 38 |  |
|  | **Mean (SD)** | 290.7 (189.86) | 272.4 (167.84) | 0.6609 |
|  | **Median (Q1─Q3)** | 244.5  (114.0─444.5) | 243.0  (121.0─392.0) |  |
| **Cockroach** | |  |  |  |
| **Positive** | **Number of subject, n (non-missing)** | 35 | 28 |  |
|  | **Mean (SD)** | 319.1 (168.77) | 356.2 (189.01) | 0.4136 |
|  | **Median (Q1─Q3)** | 328.0  (188.0- 437.0) | 341.5  (193.5- 494.5) |  |
| **Negative** | **Number of subject, n (non-missing)** | 92 | 89 |  |
|  | **Mean (SD)** | 265.0 (191.67) | 272.1 (177.55) | 0.7943 |
|  | **Median (Q1─Q3)** | 184.5  (117.5─407.5) | 239.0  (131.0─423.0) |  |
| **Dog dander** | |  |  |  |
| **Positive** | **Number of subject, n (non-missing)** | 31 | 33 |  |
|  | **Mean (SD)** | 330.1 (187.04) | 314.4 (190.74) | 0.7398 |
|  | **Median (Q1─Q3)** | 328.0  (181.0─482.0) | 282.0  (149.0─479.0) |  |
| **Negative** | **Number of subject, n (non-missing)** | 96 | 84 |  |
|  | **Mean (SD)** | 263.6 (184.46) | 283.6 (180.45) | 0.4659 |
|  | **Median (Q1─Q3)** | 189.0  (114.0─407.5) | 243.0  (131.0─448.0) |  |
| **Perennial allergic rhinitis** | |  |  |  |
| **Positive** | **Number of subject, n (non-missing)** | 38 | 38 |  |
|  | **Mean (SD)** | 328.9 (207.88) | 348.2 (193.99) | 0.6778 |
|  | **Median (Q1─Q3)** | 319.0  (138.0─520.0) | 343.5  (148.0─495.0) |  |
| **Negative** | **Number of subject, n (non-missing)** | 98 | 100 |  |
|  | **Mean (SD)** | 265.4 (177.63) | 281.4 (174.47) | 0.5227 |
|  | **Median (Q1─Q3)** | 226.0  (119.0─ 374.0) | 244.0  (136.5─ 415.0) |  |
| **AQLQ** | | | | |
| **Number of allergens** | |  |  |  |
| **1** | **Number of subject, n (non-missing)** | 20 | 36 |  |
|  | **Mean (SD)** | 289.5 (233.79) | 279.1 (195.92) | 0.8598 |
|  | **Median (Q1─Q3)** | 218.0  (84.5 465.0) | 229.5  (108.0─415.0) |  |
| **2** | **Number of subject, n (non-missing)** | 37 | 35 |  |
|  | **Mean (SD)** | 256.2 (178.17) | 291.6 (174.21) | 0.3967 |
|  | **Median (Q1─Q3)** | 185.0  (111.0─376.0) | 291.0  (134.0─429.0) |  |
| **3** | **Number of subject, n (non-missing)** | 22 | 10 |  |
|  | **Mean (SD)** | 261.3 (177.96) | 321.0 (221.18) | 0.4210 |
|  | **Median (Q1─Q3)** | 182.5  (122.0─428.0) | 247.0  (139.0─453.0) |  |
| **>3** | **Number of subject, n (non-missing)** | 40 | 33 |  |
|  | **Mean (SD)** | 315.5 (183.07) | 353.6 (165.20) | 0.3583 |
|  | **Median (Q1─Q3)** | 305.0  (159.5─441.5) | 384.0  (221.0─487.0) |  |
| ***Dermatophagoides farinae*** | |  |  |  |
| **Positive** | **Number of subject, n (non-missing)** | 85 | 65 |  |
|  | **Mean (SD)** | 282.6 (193.40) | 289.2 (187.24) | 0.8337 |
|  | **Median (Q1─Q3)** | 231.0  (121.0─440.0) | 242.0  (134.0─472.0) |  |
| **Negative** | **Number of subject, n (non-missing)** | 25 | 29 |  |
|  | **Mean (SD)** | 273.6 (176.61) | 309.5 (184.46) | 0.4706 |
|  | **Median (Q1─Q3)** | 211.0  (117.0─376.0) | 291.0  (141.0─455.0) |  |
| ***Dermatophagoides pteronyssinus*** | |  |  |  |
| **Positive** | **Number of subject, n (non-missing)** | 79 | 61 |  |
|  | **Mean (SD)** | 272.0 (185.39) | 321.4 (192.59) | 0.1264 |
|  | **Median (Q1─Q3)** | 221.0  (121.0─386.0) | 255.0  (160.0─479.0) |  |
| **Negative** | **Number of subject, n (non-missing)** | 31 | 33 |  |
|  | **Mean (SD)** | 302.4 (199.23) | 247.5 (164.17) | 0.2326 |
|  | **Median (Q1─Q3)** | 300.0  (111.0─460.0) | 175.0  (112.0─371.0) |  |
| **Cockroach** | |  |  |  |
| **Positive** | **Number of subject, n (non-missing)** | 28 | 21 |  |
|  | **Mean (SD)** | 317.1 (164.52) | 393.0 (180.78) | 0.1319 |
|  | **Median (Q1─Q3)** | 334.0  (182.0─435.0) | 455.0  (254.0─495.0) |  |
| **Negative** | **Number of subject, n (non-missing)** | 82 | 73 |  |
|  | **Mean (SD)** | 268.1 (195.99) | 267.4 (178.48) | 0.9818 |
|  | **Median (Q1─Q3)** | 182.5  (117.0─440.0) | 214.0  (131.0─394.0) |  |
| **Dog dander** | |  |  |  |
| **Positive** | **Number of subject, n (non-missing)** | 28 | 28 |  |
|  | **Mean (SD)** | 314.8 (182.03) | 328.6 (193.12) | 0.7839 |
|  | **Median (Q1─Q3)** | 293.5  (178.5─409.5) | 288.0  (162.0─486.5) |  |
| **Negative** | **Number of subject, n (non-missing)** | 82 | 66 | 0.6858 |
|  | **Mean (SD)** | 268.8 (190.95) | 281.4 (182.03) |  |
|  | **Median (Q1─Q3)** | 186.5  (111.0─437.0) | 243.0  (131.0─453.0) |  |
| **Perennial allergic rhinitis** | |  |  |  |
| **Positive** | **Number of subject, n (non-missing)** | 36 | 34 |  |
|  | **Mean (SD)** | 324.1 (206.21) | 346.9 (192.32) | 0.6344 |
|  | **Median (Q1─Q3)** | 307.0  (145.0─494.5) | 343.5  (160.0─479.0) |  |
| **Negative** | **Number of subject, n (non-missing)** | 83 | 80 |  |
|  | **Mean (SD)** | 264.7 (179.99) | 291.7 (178.09) | 0.3366 |
|  | **Median (Q1─Q3)** | 201.0  (117.0─376.0) | 252.5  (135.5─432.5) |  |
| **Investigator-GETE** | | | | |
| **Number of allergens** | |  |  |  |
| **1** | **Number of subject, n (non-missing)** | 40 | 60 |  |
|  | **Mean (SD)** | 309.9 (206.73) | 297.5 (192.81) | 0.7610 |
|  | **Median (Q1─Q3)** | 262.5  (147.0─468.5) | 249.5  (131.0─478.0) |  |
| **2** | **Number of subject, n (non-missing)** | 59 | 60 |  |
|  | **Mean (SD)** | 248.4 (170.68) | 274.8 (164.41) | 0.3923 |
|  | **Median (Q1─Q3)** | 188.0  (111.0─376.0) | 242.5  (134.5─381.5) |  |
| **3** | **Number of subject, n (non-missing)** | 30 | 25 |  |
|  | **Mean (SD)** | 280.9 (194.15) | 256.7 (217.81) | 0.6652 |
|  | **Median (Q1─Q3)** | 187.0  (122.0─428.0) | 154.0  (80.0─423.0) |  |
| **>3** | **Number of subject, n (non-missing)** | 59 | 49 |  |
|  | **Mean (SD)** | 316.9 (181.35) | 346.7 (174.65) | 0.3898 |
|  | **Median (Q1─Q3)** | 300.0  (143.0─443.0) | 344.0  (172.0─487.0) |  |
| ***Dermatophagoides farinae*** | |  |  |  |
| **Positive** | **Number of subject, n (non-missing)** | 127 | 107 |  |
|  | **Mean (SD)** | 284.3 (191.21) | 292.1 (188.27) | 0.7565 |
|  | **Median (Q1─Q3)** | 253.0  (120.0─433.0) | 242.0  (134.0─477.0) |  |
| **Negative** | **Number of subject, n (non-missing)** | 48 | 55 |  |
|  | **Mean (SD)** | 283.5 (171.68) | 290.0 (183.74) | 0.8535 |
|  | **Median (Q1─Q3)** | 250.0  (147.0─401.0) | 242.0  (138.0─443.0) |  |
| ***Dermatophagoides pteronyssinus*** | |  |  |  |
| **Positive** | **Number of subject, n (non-missing)** | 123 | 106 |  |
|  | **Mean (SD)** | 279.8 (184.07) | 302.3 (191.13) | 0.3653 |
|  | **Median (Q1─Q3)** | 249.0  (121.0─408.0) | 245.5  (143.0─478.0) |  |
| **Negative** | **Number of subject, n (non-missing)** | 52 | 56 |  |
|  | **Mean (SD)** | 294.3 (190.52) | 270.7 (176.23) | 0.5043 |
|  | **Median (Q1─Q3)** | 254.0  (118.0─456.0) | 232.5  (114.0─417.5) |  |
| **Cockroach** | |  |  |  |
| **Positive** | **Number of subject, n (non-missing)** | 46 | 39 |  |
|  | **Mean (SD)** | 319.0 (174.26) | 362.1 (200.91) | 0.3009 |
|  | **Median (Q1─Q3)** | 312.0  (188.0─443.0) | 341.5  (163.0─546.5) |  |
| **Negative** | **Number of subject, n (non-missing)** | 129 | 126 |  |
|  | **Mean (SD)** | 271.7 (188.54) | 271.2 (177.45) | 0.9818 |
|  | **Median (Q1─Q3)** | 201.0  (119.0─408.0) | 229.0  (131.0─436.0) |  |
| **Dog dander** | |  |  |  |
| **Positive** | **Number of subject, n (non-missing)** | 41 | 39 |  |
|  | **Mean (SD)** | 303.8 (182.69) | 310.1 (191.83) | 0.8794 |
|  | **Median (Q1─Q3)** | 260.0  (148.0─408.0) | 282.0  (148.0─479.0) |  |
| **Negative** | **Number of subject, n (non-missing)** | 134 | 123 |  |
|  | **Mean (SD)** | 278.1 (186.71) | 285.4 (184.75) | 0.7521 |
|  | **Median (Q1─Q3)** | 237.0  (118.0─428.0) | 239.0  (131.0─455.0) |  |
| **Perennial allergic rhinitis** | |  |  |  |
| **Positive** | **Number of subject, n (non-missing)** | 54 | 60 |  |
|  | **Mean (SD)** | 335.8 (203.40) | 323.0 (197.25) | 0.7329 |
|  | **Median (Q1─Q3)** | 308.5  (152.0─491.0) | 299.0  (145.0─487.0) |  |
| **Negative** | **Number of subject, n (non-missing)** | 134 | 134 |  |
|  | **Mean (SD)** | 269.0 (176.76) | 286.3 (178.36) | 0.4249 |
|  | **Median (Q1─Q3)** | 237.0  (120.0─374.0) | 243.0  (136.0─443.0) |  |
| **Patient-GETE** | | | | |
| **Number of allergens** | |  |  |  |
| **1** | **Number of subject, n (non-missing)** | 40 | 60 |  |
|  | **Mean (SD)** | 309.9 (206.73) | 297.5 (192.81) | 0.7610 |
|  | **Median (Q1─Q3)** | 262.5  (147.0─468.5) | 249.5  (131.0─478.0) |  |
| **2** | **Number of subject, n (non-missing)** | 59 | 59 |  |
|  | **Mean (SD)** | 248.4 (170.68) | 277.8 (164.14) | 0.3424 |
|  | **Median (Q1─Q3)** | 188.0  (111.0─376.0) | 244.0  (135.0─392.0) |  |
| **3** | **Number of subject, n (non-missing)** | 30 | 25 |  |
|  | **Mean (SD)** | 280.9 (194.15) | 256.7 (217.81) | 0.6652 |
|  | **Median (Q1─Q3)** | 187.0  (122.0─428.0) | 154.0  (80.0─423.0) |  |
| **>3** | **Number of subject, n (non-missing)** | 59 | 49 |  |
|  | **Mean (SD)** | 316.9 (181.35) | 346.7 (174.65) | 0.3898 |
|  | **Median (Q1─Q3)** | 300.0  (143.0─443.0) | 344.0  (172.0─487.0) |  |
| ***Dermatophagoides farinae*** | |  |  |  |
| **Positive** | **Number of subject, n (non-missing)** | 127 | 107 |  |
|  | **Mean (SD)** | 284.3 (191.21) | 292.1 (188.27) | 0.7565 |
|  | **Median (Q1─Q3)** | 253.0  (120.0─433.0) | 242.0  (134.0─477.0) |  |
| **Negative** | **Number of subject, n (non-missing)** | 48 | 54 |  |
|  | **Mean (SD)** | 283.5 (171.68) | 293.6 (183.52) | 0.7757 |
|  | **Median (Q1─Q3)** | 250.0  (147.0─401.0) | 243.0  (141.0─443.0) |  |
| ***Dermatophagoides pteronyssinus*** | |  |  |  |
| **Positive** | **Number of subject, n (non-missing)** | 123 | 106 |  |
|  | **Mean (SD)** | 279.8 (184.07) | 302.3 (191.13) | 0.3653 |
|  | **Median (Q1─Q3)** | 249.0  (121.0─408.0) | 245.5  (143.0─478.0) |  |
| **Negative** | **Number of subject, n (non-missing)** | 52 | 55 |  |
|  | **Mean (SD)** | 294.3 (190.52) | 273.8 (176.25) | 0.5646 |
|  | **Median (Q1─Q3)** | 254.0  (118.0─456.0) | 239.0  (116.0─443.0) |  |
| **Cockroach** | |  |  |  |
| **Positive** | **Number of subject, n (non-missing)** | 46 | 35 |  |
|  | **Mean (SD)** | 319.0 (174.26) | 369.7 (198.56) | 0.2252 |
|  | **Median (Q1─Q3)** | 312.0  (188.0─443.0) | 344.0  (172.0─552.0) |  |
| **Negative** | **Number of subject, n (non-missing)** | 129 | 126 |  |
|  | **Mean (SD)** | 271.7 (188.54) | 271.2 (177.45) | 0.9818 |
|  | **Median (Q1─Q3)** | 201.0  (119.0─408.0) | 229.0  (131.0─436.0) |  |
| **Dog dander** | |  |  |  |
| **Positive** | **Number of subject, n (non-missing)** | 41 | 39 |  |
|  | **Mean (SD)** | 303.8 (182.69) | 310.1 (191.83) | 0.8794 |
|  | **Median (Q1─Q3)** | 260.0  (148.0─408.0) | 282.0  (148.0─479.0) |  |
| **Negative** | **Number of subject, n (non-missing)** | 134 | 122 |  |
|  | **Mean (SD)** | 278.1 (186.71) | 287.0 (184.71) | 0.7028 |
|  | **Median (Q1─Q3)** | 237.0  (118.0─428.0) | 240.5  (134.0- 455.0) |  |
| **Perennial allergic rhinitis** | |  |  |  |
| **Positive** | **Number of subject, n (non-missing)** | 54 | 60 |  |
|  | **Mean (SD)** | 335.8 (203.40) | 323.0 (197.25) | 0.7329 |
|  | **Median (Q1─Q3)** | 308.5  (152.0- 491.0) | 299.0  (145.0- 487.0) |  |
| **Negative** | **Number of subject, n (non-missing)** | 134 | 133 |  |
|  | **Mean (SD)** | 269.0 (176.76) | 287.8 (178.27) | 0.3887 |
|  | **Median (Q1─Q3)** | 237.0  (120.0- 374.0) | 244.0  (137.0- 443.0) |  |

Full analyses set. P-value compared mean of baseline IgE between omalizumab and placebo group.

ACQ, Asthma Control Questionnaire; AQLQ, Asthma Quality of Life Questionnaire; CI, confidence interval; GETE, Global Evaluation of Treatment Effectiveness; N, total number of patients; n, number of patients; OMA, omalizumab; PAR, perennial allergic rhinitis; PBO, placebo; SD, standard deviation

**Supplemental Appendix 7:** Total (A) and free (B) IgE of the PK/PD patients at baseline, Week 1, and Week 24

**
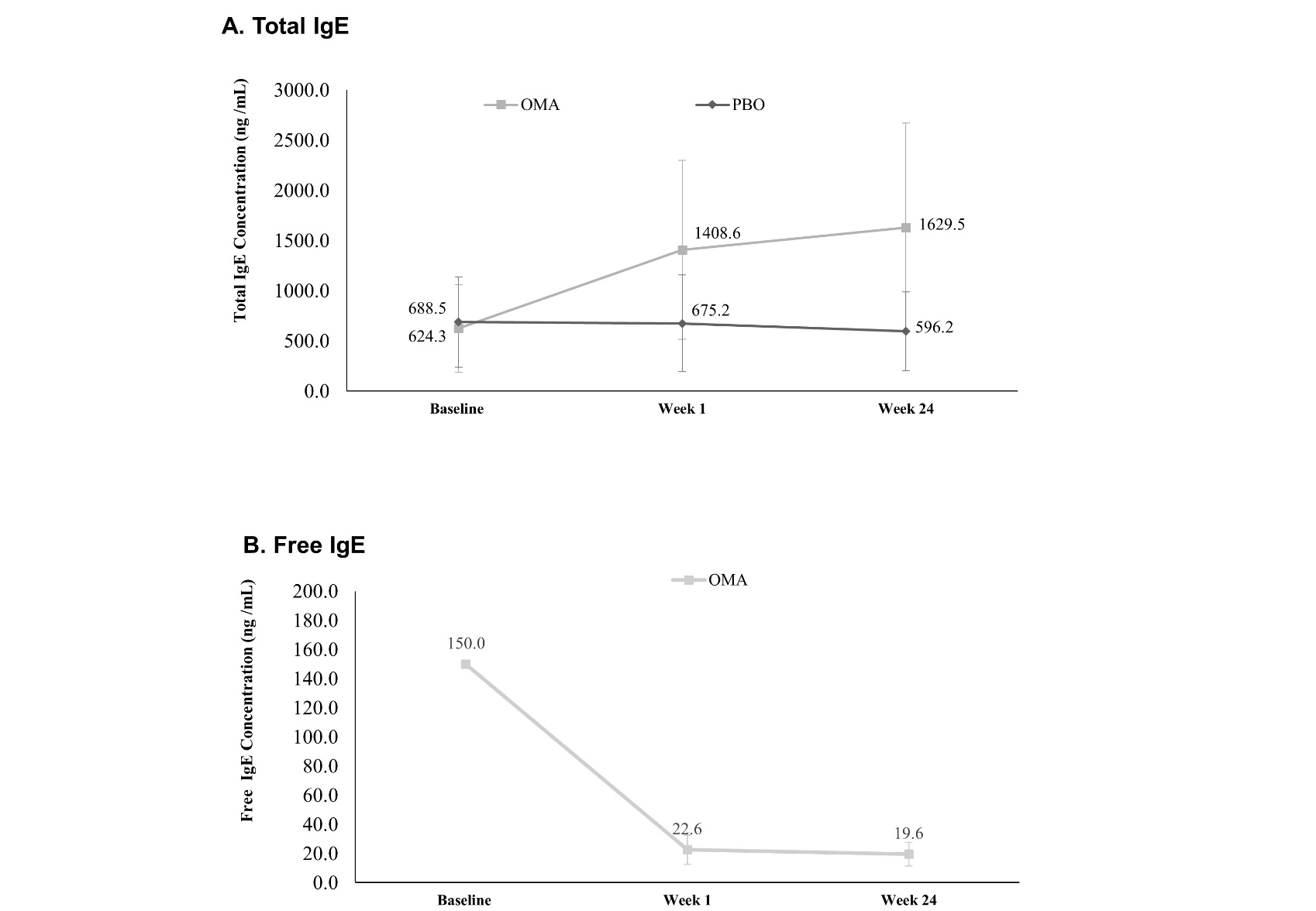
**

Values of free IgE above the ULOQ (150 ng/mL [62.5 IU/mL]) at baseline were set to 150 ng/mL (62.5 IU/mL). IgE, immunoglobulin E; OMA, omalizumab; PBO, placebo; ULOQ, upper limit of quantification
